# Association between calcitonin receptor gene polymorphisms and calcium stone urolithiasis: A meta-analysis

**DOI:** 10.1590/S1677-5538.IBJU.2019.0061

**Published:** 2019-01-29

**Authors:** Jinchun Xing, Jiaxuan Qin, Zonglong Cai, Bo Duan, Peide Bai

**Affiliations:** 1 Department of Urology Surgery Affiliated Hospital Xiamen University China Department of Urology Surgery, the First Affiliated Hospital of Xiamen University; Center of Diagnosis and Treatment of Urinary System Diseases, the First Affiliated Hospital of Xiamen University; the Key Laboratory of Urinary Tract Tumors and Calculi of Xiamen City, the First Affiliated Hospital of Xiamen University. Xiamen, Fujian, China;; Center of Diagnosis and Treatment of Urinary System Diseases Affiliated Hospital Xiamen University China; Laboratory of Urinary Tract Tumors and Calculi of Xiamen City Affiliated Hospital Xiamen University Xiamen Fujian China; 2 Clinical Medical School Fujian Medical University Xiamen Fujian China The First Clinical Medical School of Fujian Medical University. Xiamen, Fujian, China

**Keywords:** Receptors, Calcitonin, Urolithiasis, Meta-Analysis [Publication Type]

## Abstract

**Purpose:**

It has been reported that calcitonin receptor (CALCR) gene polymorphisms might be associated with calcium stone urolithiasis. Owing to mixed and inconclusive results, we conducted a meta-analysis to summarize and clarify this association.

**Materials and Methods:**

A systematic search of studies on the association between CALCR gene polymorphisms and calcium stone urolithiasis susceptibility was conducted in databases.

**Results:**

Odds ratios and 95% confidence intervals were used to pool the effect size. Five articles were included in our meta-analysis.

**Conclusions:**

CALCR rs1801197 might be associated with increased risk of calcium stone urolithiasis. There is insufficient data to fully confirm the association between CALCR rs1042138 and calcium stone urolithiasis susceptibility. Well-designed studies with larger sample size and more subgroups are required to validate the risk identified in the current meta-analysis.

## INTRODUCTION

Urolithiasis is a relatively common health problem which is likely associated with the effects of multiple genes in combination with lifestyles and environmental factors (1, 2). Majority of urolithiasis is calcium stone. The calcitonin receptor (CALCR) is a 7-pass transmembrane G-protein-coupled receptor which reacts in response to the calcium metabolism-related hormone calcitonin (3). By binding calcitonin receptor on the osteoclasts in the bone and the renal tubular cells, calcitonin causes inhibition of bone resorption and lowers serum calcium concentration (4). Therefore, calcitonin receptor gene polymorphisms might cause calcium metabolic disorders. Some calcitonin receptor gene polymorphisms, like SNP rs1801197 (1377C>T) and rs1042138 (3’UTR+18C>T), have shown association with bone mineral density (5, 6). The SNP rs1801197 alters the encoded amino acid from proline to leucine (7). Researchers have also investigated the association of those SNPs with urolithiasis.

Association between calcitonin receptor gene polymorphisms and calcium stone urolithiasis susceptibility has been studied in several populations. Sample sizes in these studies are relatively small. Therefore, we decided to perform a meta-analysis to estimate it.

## MATERIALS AND METHODS

### Identification of eligible studies

A systematic search in Pubmed by Medline, Embase, Cochrane Library, clinicaltrials.gov, CNKI (China National Knowledge Infrastructure) databases were carried out by two independent investigators. The following terms were used: “calcitonin Receptor OR CTR OR CALCR” AND “stone OR calculus OR calculi OR lithiasis OR Nephrolithiasis OR urolithiasis” AND “polymorphisms OR polymorphism”, without any limitation applied. The last search update was performed on August 3, 2017. References of related studies and reviews were also manually searched for additional studies.

### Inclusion and exclusion criteria

Studies selected in this meta-analysis should have met the following inclusion criteria: (1) evaluation of the association between calcitonin receptor gene polymorphisms and calcium stone urolithiasis susceptibility; (2) case-control study; (3) studies focusing on tissues of human beings; (4) detailed genotype data could be acquired to calculate the odds ratios (ORs) and 95% confidence intervals (95% CIs). Exclusion criteria: (1) duplication of previous publications (when there were multiple publications from the same population, only the largest study was included); (2) comment, review and editorial; (3) study without detailed genotype data; (4) GWAS; (5) studies focusing on cell lines. Dissertation thesis were included in the analysis.

Study selection was achieved by two investigators independently, according to the inclusion and exclusion criteria by screening the title, abstract and full-text. Any dispute was solved by discussion.

### Data extraction

Two investigators extracted data of the eligible studies independently. In the case of a conflict, an agreement was reached by discussion. If the dissent still existed, the third investigator would be involved to adjudicate the disagreements. Try to contact the author by email for detailed genotype data.

The following contents were collected: first author’s surname, year of publication, chemical composition of urinary calculi, the characteristics of cases and controls, source of control groups, country of origin, the detective sample, ethnicity, genotyping method, Hardy-Weinberg equilibrium, number of cases and controls for each genotype.

### Methodological quality assessment

The qualities of included studies were evaluated independently by two investigators according to Newcastle-Ottawa Scale (NOS) (8) and the most important factor was ”age, gender and country”. Quality scores range from 0 to 9, and higher scores meant better quality of the study. Disagreement was resolved through discussion.

### Statistics analysis

Our meta-analysis was conducted according to the PRISMA checklists (9). Hardy-Weinberg equilibrium (HWE) was evaluated for each study by Chi-square test in control groups, and P <0.05 was considered as a significant departure from HWE. OR and 95% CIs were calculated to evaluate the strength of the association between calcitonin receptor gene polymorphisms and calcium stone urolithiasis susceptibility. Pooled ORs were obtained from combination of single studies by allelic comparison (T vs. C), dominant model (CT+TT vs. CC), recessive model (TT vs. CC+CT), homozygote comparison (TT vs. CC) and heterozygote comparison (CT vs. CC), respectively. The statistical significant level was determined by Z-test with P value less than 0.05.

Heterogeneity was evaluated by Q-test and I ^2^ index (10). When Q-test’s P-value was less than 0.10 and/or I ^2^ index was more than 50%, the random-effects model (DerSimonian and Laird method) was used; otherwise, the fixed-effects model (Mantel and Haenszel method) was conducted (11).

Sensitivity analyses were performed towards each genetic model to evaluate effect of each study on combined ORs by sequentially excluding each study in total and in any subgroup including more than two studies. Potential publication bias was checked by Begg’s funnel (12) plots and Egger’s test (13). An asymmetric plot, the P value of Begg’s test (P _B_ ) less than 0.05, and the P value of Egger’s test (P _E_ ) less than 0.05 was considered a significant publication bias. All statistical analyses were performed with Stata 12.0 software (StataCorp, College Station, Texas, USA). A two-tailed P <0.05 was considered significant except for specified conditions, where a certain P value was declared.

## RESULTS

### Characteristics of studies

**Figure 1:**
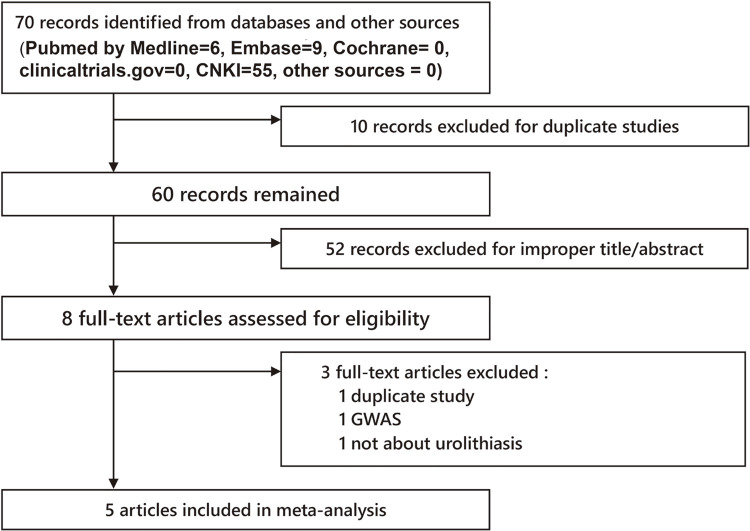
Flow Chart of study selection.

A total of 70 articles were acquired from databases (Pubmed by Medline=6, Embase=9, Cochrane=0, clinicaltrials.gov=0, CNKI=55, other sources (from manually search) =0). The selection process is shown in Figure-1. Three full-text articles were excluded (1 duplicate study (14), 1 GWAS (15), 1 not about urolithiasis (16)). Finally, 5 articles (17-21) were included in our meta-analysis. The characteristics of each study are shown in Table-1 and Table-2. Different genotyping methods were utilized including PCR-RFLP, PCR-SSCP and Sequencing. Blood samples were used for genotyping in all studies. The control group of study NO 1.4, 1.5 and 1.5.1 had shown significant departure from HWE.

**Table 1:** Characteristics of studies included in the meta-analysis.

NO.	Study ID	Year	Country or Area	Ethnicity	Control Type	Genotyping Method	Case			Control			P for HWE*	Quality
	CALCR rs1801197						TT	CT	CC	TT	CT	CC		
1.1	**Bid HK (17)**	2005	Northern India	Indian	PB*	PCR-RFLP	10	24	16	4	26	30	0.604	9
1.2	**Song GL (18)**	2014	China	Uyghur	PB	PCR-RFLP	4	23	62	0	21	100	0.296	9
1.3	**Chen WC (19)**	2001	Taiwan	Asian	PB	PCR-RFLP	2	25	75	0	6	99	0.763	7
1.3.1	Male						2	18	56	0	0	60	NA*	
1.3.2	Female						0	7	19	0	6	39	0.632	
1.4	**Shakhssalim N (20)**	2014	Iran	Caucasian	PB	PCR-SSCP	17	47	37	17	38	51	0.039*	8
1.5	**Mitra P (21)**	2017	West India	Indian	PB	Sequencing	24	78	50	6	69	69	0.026	9
1.5.1	Male						16	59	37	2	47	56	0.026	
1.5.2	Female						8	19	13	3	22	14	0.159	
	**CALCR rs1042138**						TT	CT	CC	TT	CT	CC		
2.1	**Shakhssalim N (20)**	2014	Iran	Caucasian	PB	PCR-SSCP	1	27	73	0	0	101	NA	8
2.2	**Mitra P (21)**	2017	West India	Indian	PB	Sequencing	6	51	95	2	49	93	0.110	9
2.2.1	Male						6	40	66	2	34	69	0.344	
2.2.2	Female						0	11	29	0	15	24	0.137	

* HWE: Hardy–Weinberg equilibrium; **PB:** population-based; **NA:** not available.

* Results with statistical significant difference were marked as bold.

**Table 2:** Characteristics of cases and controls.

Study ID	Case	Control
**Bid HK (17)**	50 **pediatric patients** (age range 2–14 years) with renal stones from the Northern Indian states of Uttar Pradesh and Bihar. Patients who had history of bowel disease, renal tubular acidosis and urinary tract anomalies were excluded. Stone composition was verified using Xray crystallography. **38 patients had sufficient specimens available for stone composition analysis: 64% were whewellite, 28% whewellite and weddellite, and 8% whewellite and uricite.**	60 healthy children (age range 4–16 years) who had no history of stone disease were drawn from the general population taking care to match age, socioeconomic status, dietary habits, religion and gender.
**Song GL (18)**	89 **pediatric patients** (age range 0.5–7 years) with upper urinary tract calculi from southern Xinjiang of China. Patients who had rickets, thyroid dysfunction, parathyroid dysfunction, symptomatic urinary tract infection, ureteropelvic junction obstruction, renal insufficiency, renal tubular acidosis, tumor, osteoporosis, taking vitamin D or calcium supplements were. All stone composition was **calcium stone** , such as calcium oxalate, calcium phosphate, calcium carbonate, etc.	121 healthy children (same age range) who had no history of stone disease were drawn from the general population of southern Xinjiang taking care to match age, gender, socioeconomic status, dietary habits and religion.
**Chen WC (19)**	102 **adult patients** (age range 23–76 years) with **recurrent calcium oxalate stones** who had been treated in the department of urology were included. Patients who showed symptoms of urinary tract infections during the period of stone treatment were excluded. Stone composition was verified by infrared spectroscopy revealing calcium oxalate monohydrate, dihydrate, or a combination of the two.	105 healthy volunteers (age range 40–87 years) with no familial history of stone disease, or renal calcification (following renal ultrasonography tests, as well as routine tests made from urinary microscopic hematuria).
**Shakhssalim N (20)**	105 **adult men** (age range 30–55 years) with a history of **recurrent calcium urinary stones** who had at least two recurrent episodes during the past 5 years. Patients with histories of known metabolic, gastrointestinal, hepatic, renal or endocrinological diseases, with any anatomic abnormality or obstruction in the urinary tract, or taking any drugs which may affect urine composition were excluded.	101 adult men (age range 30–55 years) were selected from volunteers who had been referred to the Ophthalmology Clinic of the Labbafinejad hospital, or unrelated healthy friends of the patients who did not express any personal or family history of urolithiasis. Ultrasonographic examination was performed and men with any evidence of urolithiasis were excluded. Other exclusion criteria were similar to those for the case group.
**Mitra P (21)**	152 **adult patients** (age range 18–75 years) with **calcium containing renal stone(s)** in Kolkata, West Bengal, India. Patients with histories of known metabolic, gastrointestinal, renal, endocrinological disorders or patients taking any drugs like steroids, diuretics were excluded. The composition of stone was analyzed by chemical tests. Only patients with calcium containing kidney stones were included.	144 age and sex matched healthy individuals from the same geographical region and socioeconomic status who was negative in family history for kidney stone. Ultrasonographic examination was performed to confirm no evidence of renal stone.

### Overall analyses and Subgroup analyses

Summary results of each genetic model are listed in Table-3. In pediatric urolithiasis subgroup and overall, significantly increased risk of calcium stone urolithiasis was found in CALCR rs1801197 in all genetic models. In adult urolithiasis and its male subgroup, significantly increased risk of calcium stone urolithiasis was found in CALCR rs1801197 in heterozygote comparison (CT vs. CC) and dominant model (CT+TT vs. CC). In adult urolithiasis, significantly increased risk of calcium stone urolithiasis was also found in CALCR rs1801197 in allelic comparison (T vs. C). No statistically significant changes of calcium stone urolithiasis risk was found in other analyses.

**Table 3:** Summary of pooled ORs in the meta-analysis.

	Number	T vs C		TT vs CC		CT vs CC		CT+TT vs CC		TT vs CC+CT	
CALCR rs1801197	(cases/controls)	OR*(95%CI*)	I2(%)	OR(95%CI)	I2(%)	OR(95%CI)	I2(%)	OR(95%CI)	I2(%)	OR(95%CI)	I2(%)
**Overall** (1.1*,1.2,1.3,1.4,1.5)	494/536	1.987(1.401-2.819)*	**56.9**	**3.163(1.895-5.279)**	43.5	**1.910(1.433-2.544)**	30.7	**2.117(1.608-2.787)**	32.2	2.805(1.195-6.587)*	**51.0**
PU* (1.1, 1.2)	139/181	**2.094(1.394-3.145)**	0.0	**6.140(1.895-19.89)**	0.0	**1.752(1.042-2.947)**	0.0	**2.095(1.270-3.456)**	0.0	**4.631(1.527-14.04)**	0.0
AU* (1.3, 1.4, 1.5)	355/355	**2.052(1.141-3.690)**	**76.9**	2.955(0.937-9.315)	**61.3**	**2.188(1.161-4.126)**	**64.9**	**2.329(1.255-4.323)**	**66.1**	2.305(0.708-7.500)	**66.2**
**Male of AU** (1.3.1, 1.4, 1.5.1)	289/271	2.036(0.963-4.305)	**76.3**	3.862(0.724-20.61)	**70.0**	**2.234(1.011-4.935)**	**61.2**	**2.437(1.063-5.586)**	**67.1**	2.855(0.557-14.64)	**70.1**
**Female of AU** (1.3.2, 1.5.2)	66/84	1.541(0.881-2.694)	0.0	NA*	NA	1.335(0.627-2.840)	29.1	1.509(0.722-3.154)	0.0	NA	NA
ARU* (1.3, 1.4)	203/211	2.557(0.605-10.81)	**88.4**	1.584(0.746-3.366)	0.0	2.891(0.918-9.101)	**76.5**	2.920(0.808-10.55)	**82.2**	1.201(0.595-2.421)	1.0
**Male of ARU** (1.3.1, 1.4)	177/166	5.796(0.141-239.1)	**85.7**	1.552(0.728-3.307)	0.0	6.349(0.218-184.6)	**82.0**	6.576(0.184-234.4)	**84.1**	1.173(0.580-2.371)	0.0
**CALCR rs1042138**											
**AU** (2.1, 2.2)	253/245	7.502(0.067-843.2)	**91.1**	3.150(0.738-13.43)	0.0	7.477(0.049-1144)	**92.0**	7.871(0.054-1144)	**91.8**	2.940(0.694-12.46)	0.0
**Male of AU** (2.1, 2.2.1)	213/206	8.130(0.084-787.7)	**90.4**	3.321(0.773-14.26)	0.0	8.191(0.064-1041)	**91.3**	8.663(0.073-1029)	**91.1**	2.938(0.690-12.51)	0.0

*OR: Odds ratio; CI: confidence interval; PU: Pediatric Urolithiasis; AU: Adult Urolithiasis; ARU: adult recurrent urolithiasis; NA: not available.

*NO of studies included in the meta-analysis.

*Results with statistical significant difference were marked as bold. Unstable results in sensitivity analyses were marked as italic. Less than three studies were included in PU, Female of AU, ARU, Male of ARU subgroup of rs1801197, and AU, Male of AU subgroup of rs1042138, so that sensitivity analyses could not be performed.

### Sensitivity analyses

Sensitivity analyses were performed in any comparison and any subgroup including more than two studies. In adult urolithiasis and its male subgroup, when study NO 1.4 or 1.5 was excluded, statistically different results were obtained in all genetic models of CALCR rs1801197. Overall, when study NO 1.1 or 1.5 was excluded, statistically different results were obtained in recessive model (TT vs. CC+CT) of CALCR rs1801197 (Table-3).

Less than three studies were included in PU, Female of AU, ARU, Male of ARU subgroup of rs1801197, and AU, Male of AU subgroup of rs1042138, so that sensitivity analyses could not be performed.

Other results showed stability in sensitivity analyses (Table-3).

### Publication bias

Begg’s funnel plot and Egger’s test were used to assess the publication bias. Symmetry of funnel plot, P value of Begg’s test (P _B_ ) and P value of Egger’s test (P _E_ ) were evaluated overall (including studies NO 1.1, 1.2, 1.3, 1.4 and 1.5). No significant publication bias was found in Egger’s test in all genetic models of CALCR rs1801197. However, in allelic comparison (T vs. C), heterozygote comparison (CT vs. CC) and dominant model (CT+TT vs. CC) of CALCR rs1801197, study NO 1.3 extended beyond the diagonal line which represents pseudo-95% CI limits about the effect estimate in funnel plot, meanwhile, the u value=1.96 (u=z in Begg’s test) in those three genetic models. In Begg’s test, 1.96 is a critical value.

## DISCUSSION

**Figure 2:**
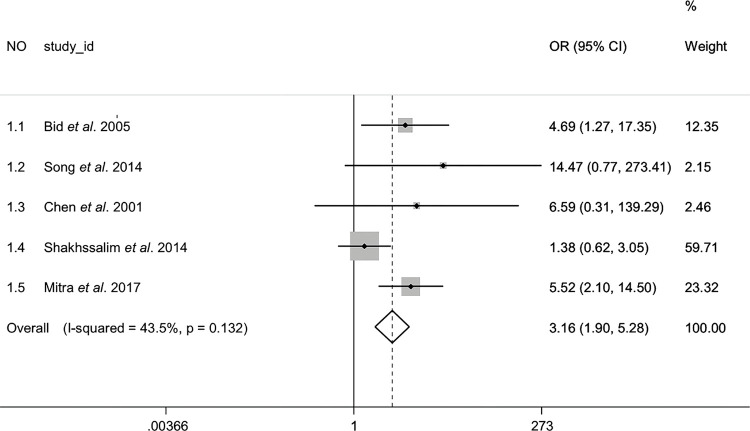
Forest plot with a fixed effect model for the association between CALCR rs1801197 and calcium stone urolithiasis in homozygote comparison (TT vs. CC). For each study, the estimate of OR and its 95% CI is plotted with a box and a horizontal line. Rhombus: pooled OR and its 95% CI.

Overall, we found CALCR rs1801197 was associated with increased risk of calcium stone urolithiasis in homozygote comparison (TT vs. CC), and the results showed stability in sensitivity analyses and no publication bias (Figure-2). Significantly increased risk was also found in other four genetic models, however, the result in recessive model (TT vs. CC+CT) lacked stability, and we got a critical value of u in Begg’s test in allelic comparison (T vs. C), heterozygote comparison (CT vs. CC) and dominant model (CT+TT vs. CC). Study NO 1.3 might play a negative role in the publication bias analyses.

In AU and its male subgroup of rs1801197, significantly increased risk was found in heterozygote comparison (CT vs. CC) and dominant model (CT+TT vs. CC). In AU, significantly increased risk was also found in allelic comparison (T vs. C) of rs1801197. However, those results lacked stability and publication bias analyses could not be performed.

In PU, Female of AU, ARU, Male of ARU subgroup of rs1801197, and AU, Male of AU subgroup of rs1042138, sensitivity analyses and publication bias analyses could not be performed.

Meanwhile, the limitations of this meta-analysis need to be addressed. To date, the number of available studies which could be included in this meta-analysis were small. Data for subgroup analyses were scanty. Sensitivity analyses and publication bias analyses could not be performed in some groups or subgroups. Studies NO 1.4, 1.5 and 1.5.1 had shown significant departure from HWE. Related studies published in other languages or unpublished were possibly missed. With those limitations, the study provided some insights on the potential association between CALCR gene polymorphims and calcium stone urolithiasis.

In conclusion, our results suggested that: CALCR rs1801197 might be associated with increased risk of calcium stone urolithiasis. There is insufficient data to fully confirm the association between CALCR rs1801197 and calcium stone urolithiasis susceptibility in pediatric urolithiasis, adult urolithiasis, adult recurrent urolithiasis subgroup and gender subgroup, and the results should be interpreted with caution. There is insufficient data to fully confirm the association between CALCR rs1042138 and calcium stone urolithiasis susceptibility, and the results should be interpreted with caution. Well-designed studies with larger sample size and more subgroups are required to validate the risk identified in the current meta-analysis.
